# Key Nutrient Intakes at Risk Among US Children in the National Health and Nutrition Examination Survey (NHANES) 2015–2016 Stratified by Age and Gender

**DOI:** 10.3390/children12020238

**Published:** 2025-02-17

**Authors:** John Lasekan, Qi Yao, Yong Choe, Grace M. Niemiro, Penni Hicks

**Affiliations:** Abbott Nutrition, Columbus, OH 43219, USA; qi.yao1@abbott.com (Q.Y.); yong.choe@abbott.com (Y.C.); grace.niemiro@abbott.com (G.M.N.); penni.hicks@abbott.com (P.H.)

**Keywords:** National Health and Nutrition Examination Survey, adequate intake, dietary reference intakes, estimated average requirement, nutrient intake gaps, children

## Abstract

Background/Objective: Inadequate nutrient intake in childhood can have lasting detrimental developmental and health outcomes. The objective of this study was to identify key nutrient intake gaps among US children. Method: Using the National Health and Nutrition Examination Survey 2015–2016 data, we compared nutrient intakes against dietary reference intakes (DRIs). Participants were grouped by age (in years): 0–0.5, 0.6–1, 1–4, 5–8, 9–14, and 15–19. Gender differences were assessed in the two older groups. Results: Linoleic acid, linolenic acid, vitamin D, and choline were the most consumed at lesser than DRIs in all age groups. Additionally, dietary fiber, vitamin A, vitamin E, vitamin K, folate, iron, and calcium were consumed at lesser than DRIs in 1–19 year olds. They also had an inadequate intake of docosahexaenoic acid (DHA). Children with nutrient intake gaps increased from infancy and toddlerhood to school-age period, with the inflection point at 5–8 years of age when parents have limited control on children’s nutrient intake. Above 9 years of age, females had greater nutrient intake gaps than males. Females in the 15–19-year-old segment especially had higher nutrient intake gaps (*p* < 0.05) for vitamin D, thiamin, riboflavin, vitamin B12, folate, iron, calcium, magnesium, phosphorus, and potassium compared to males. Selected laboratory biomarkers were used to verify the nutrient intake data. Conclusions: Key nutrient intake gaps were identified among different age groups of children in the US, which may have implications for future dietary interventions and target food formulations to help narrow these gaps.

## 1. Introduction

The population of children ages 17 years and below in the US in 2019 was estimated to be about 73 million [1]. Healthy nutritional eating pattern have been established for the US population (including children) by the U.S. Department of Agriculture, U.S. Department of Health and Human Services, Dietary Guidelines for Americans [2]. The guidelines also emphasized that the improvement of dietary shortfalls and excesses among children is important for their healthy growth and cognitive development, which will help reduce the risk of chronic disease later in life [2]. Early childhood is characterized by rapid growth and cognitive and social development. As the recommended nutrient requirements for children are higher than those for adults on a per-kilogram basis [3], the nutritional intake of children deserves special attention. As children grow older, they become more independent of their parents, and thus, they can make their own food choices, potentially leading to a less nutrient-dense dietary pattern [4,5]. An evaluation of the nutrient intake profiles across the spectrum of children’s age groups and gender could help identify nutrient intake gaps for which nutritional interventions could be developed and targeted. Such interventions could include innovative food and nutrient fortifications, nutritional education, oral supplementation, etc., tailored to different age groups with different nutritional needs.

Consequently, the primary objective of this study was to evaluate key nutrient intakes by children in the US using the NHANES 2015–2016 database and the DRI to identify nutrients consumed in less than recommended amounts (nutrient gaps). Age and gender-specific differences in nutrient intake patterns were identified and discussed. The secondary goal was to determine gender differences on identified nutrient intake gaps in the older children’s segment. In addition, the tertiary goal was to use selective laboratory biomarkers in NHANES as an objective tool to validate the nutrient intake survey data, which are sometimes subjective.

## 2. Materials and Methods

### 2.1. Study Database Source and Recommended Dietary Intake

The US National Health and Nutrition Examination Survey (NHANES) database is one of the premier nutrients and food consumption databases in the world [6]. This nationwide study is conducted every two years, and dietary intake, anthropometric values, biomarkers, or metabolic health data are available for public use. These national survey data, together with the US recommended nutrient intakes—the dietary reference intakes (DRIs), recommended dietary allowances (RDAs), adequate intakes (AIs), and estimated average requirements (EARs), published by the National Institute of Medicine [7], are useful tools in evaluating current nutritional status, identifying nutrient intake gaps, and designing and monitoring nutrition interventions in the United States. The DRIs, which were revised in 2000, encompass recommendations for between 50% and 98% of a specific age and gender group. Instead of one recommended standard, as in the 1989 RDA, the DRIs have four recommended standards (EAR, RDA, AI, and tolerable upper intake level [UL]) with different stipulated conditions for use [7]. EAR is the recommended DRI for evaluating the prevalence of inadequate nutrient intake in a group. However, EAR for some key nutrients were not set. In the absence of an EAR, the AI has been used, but with a cautionary message of overestimation of prevalence of inadequate intake [7]. Since inception, the DRIs have continuously been updated for various nutrients. DRIs for vitamin C, vitamin E, and selenium were established in 2000. The following year, vitamin A, vitamin K, copper, iron, sodium, and zinc were updated. Potassium and sodium were updated in 2005, and calcium and vitamin D were updated in 2011 [8,9,10,11,12,13]. Nutrient intake recommendations are the same for both genders during childhood with the exception that DRI are different for males and females beginning at 9 years of age.

### 2.2. Participants and Variables of Interest

Participants were part of the US National Health and Nutrition Examination Survey (NHANES) 2015–2016 cohorts between 0 and 19 years of age [14]. NHANES 2015–2016 was chosen for this study because we felt it provided more comprehensive laboratory data to match validated dietary recall data in its finality compared with subsequent data, which still have some ongoing quality control and validation, given the COVID-19 pandemic from 2019 to 2021. Children were divided into the following groups for analyses: 0–0.5, 0.6–1, 1–4, 5–8, 9–14, and 15–19 years of age. These corresponded to younger infants, older infants, toddlers, young children, preadolescents/adolescents, and teenagers, respectively. We adopted this age segmentation for this study because it represents a reasonable generalization, given the variations in school-age segmentations among different states, local educational boards in the US. For example, some states have middle schoolers, and some do not. Age groups between 9–14 and 15–19 years of age were further analyzed for gender segmentation. Dietary data were collected according to NHANES standard procedures. The Research Ethics Review Board (ERB) at the US National Center for Health Statistics (NCHS) approved the survey protocol (NCHS ERB Protocol # 2011-17), and written informed consent was obtained from all the survey participants or their proxies (for infants and younger children). Outcomes of interest included mean nutrient intakes and the proportion of children in each age and gender group not meeting the dietary reference intakes (DRIs). The DRIs comprised four standards: the recommended dietary allowances (RDA), the estimated average requirement (EAR), the adequate intake (AI), and the tolerable upper limit (UL). The RDA is the amount estimated to meet the needs of 97–98% of the target population. The EAR is the amount estimated to meet the needs of 50% of the age- and sex-specific population. It is the most appropriate for evaluating nutrient intake prevalences in groups or population but not for individuals. If there is not sufficient evidence to set an EAR, then the AI is the amount believed to be adequate for the target population [7]. The DRIs used in our current study were (EAR, AI).

### 2.3. Nutrient and Laboratory Associations

Biochemical data for the NHANES 2015–2016 cohort were assessed in this study. Laboratory procedures and sample analyses have been described in the 2015–2016 NHANES Mobile Equipment Center (MEC) Laboratory Procedures Manual [15]. These values were associated with dietary intake of nutrients by linear regression as a reliability measure due to the potential subjectivity of self-reported dietary intake. Although the numbers of laboratory markers analyzed in the 2015–2016 NHANES are extremely limited compared to previous NHANES periods, they provide a useful verification of the nutrient intake data. Laboratory values and nutrient intakes were analyzed with all children that had both values irrespective of age groups (*n* = 3310).

### 2.4. Statistical Methods

The primary variable of interest in this study was the percent of subjects within each age segment not meeting 100% of the DRI (EAR or AI) for each key nutrient. Twenty percent or greater was considered to be an important proportion of subjects not meeting the DRIs in any age group. This was based on the consensus of the authors that the threshold of at least 20% prevalence of undernutrition is a reasonable benchmark for all the nutrients, given that the important prevalence for each nutrient will vary depending on the criticality, short- and long-time impact of the nutrient, and age of the target population. For example, the impact of iron and vitamin A intake gaps might be more critical to health outcomes compared to selenium. In addition, at least 15% of children in an age group not meeting the EAR or AI for some key nutrients were noted as well. Other variables of interest evaluated in this study included intake means and standard error of the mean (SEM). The NHANES data were analyzed with SAS software (SAS^®^ Version 9.4 and SAS^®^ Enterprise Guide Version 8.3.NC) [16]. There were 9971 subjects of all ages in the NHANES 2015–2016, out of which 3489 were children from birth to 19 years of age. None of the children provided intake recall that was deemed unreliable (dr1drstz = 2); however, 179 reported consuming some breast milk (dr1drstz = 4, 0.05% of 3489, 0.02% of whole population). The 179 were kept in the whole dataset as “not missing completely at random”. Analysis was applied using the Taylor series linearization method in SAS Survey Procedures with “nomcar” option. (https://wwwn.cdc.gov/nchs/nhanes/tutorials/VarianceEstimation.aspx, accessed on 2 May 2024). This helps perform domain analysis in the domain of no missing observation. In essence, a total of 3310 (3489 -179) children from birth to 19 years of age were analyzed in terms of valid full dietary Day-1 sample and MEC day-1 weights. The children were segmented into 0–0.5 (*n* = 91), 0.5–1 (*n* = 122), 1–4 (*n* = 713), 5–8 (*n* = 676), 9–14 (*n* = 996), and 15–19 (*n* = 712) years of age. The two oldest age groups (9–14 and 15–19 years of age) were examined for each gender individually.

NHANES 2015–2016 data used in the nutrient intake gap analysis in this study included datasets obtained from Demographics Interview, Dietary Interview, dietary Day 1 total files (DR1TOT), and Laboratory Examination. Descriptive summary statistics for both the age category grouping and the age/gender category groupings were given in tables for dietary intakes using the SAS software system with appropriate weight and method to reflect the actual sampling procedures used to collect NHANES data. Day 1 weights (WTDRD1) were constructed by taking the MEC sample weights (WTMEC2YR). They were further adjusted for (a) the additional non-response and (b) the differential allocation by weekdays (Monday through Thursday), Fridays, Saturdays, and Sundays for the dietary intake data collection using the recommended procedure at (https://wwwn.cdc.gov/Nchs/Data/Nhanes/Public/2015/DataFiles/DR1TOT_I.htm, accessed on 2 May 2024). A single 24 h recall was sufficient for analyzing mean nutrient intakes from foods and beverages. The accurate estimated means and their standard errors were determined using the Taylor series linearization method and the SAS Survey Procedures (PROC SURVEYMEANS) described at (https://wwwn.cdc.gov/nchs/nhanes/tutorials/VarianceEstimation.aspx, accessed on 2 May 2024).

EAR and AI were used as the criteria for assessing the adequacy of nutrient intake.

- Percent of criteria met = actual daily intake/recommended daily intake (EAR or AI) × 100.

If the percent of criteria met was ≥100% (i.e., either met or exceeded the recommendation), it was classified as having met the recommendation; otherwise, it was classified as not meeting the recommendation. Once the adequacy of intake was assessed, descriptive statistics were calculated for each of the age categories and each of the age/gender categories for both the continuous parameter of percent of criteria met and the categorical parameter of adequacy of intake. Descriptive statistics were displayed for each nutrient separately for the EAR and AI recommendations. For docosahexaenoic acid (DHA) and arachidonic acid (ARA), only the mean intakes were listed because they did not have DRIs (EAR or AI) in the US. Their intake gaps were not assessed. Gender differences in the two older age groups were analyzed based on two sample t-test at *p* < 0.05.

The association of available laboratory (blood) biomarkers with selected nutrient intakes in NHANES 2015–2016 data were assessed using simple regression model via statement of PROC SURVEYREG with weight of “wtdrd1”. Laboratory variables in blood and Natural Log-transformed laboratory variables in blood were used as responses in the model to determine the association with the selected nutrient intakes.

## 3. Results

### 3.1. Demographics of Subjects

Selected demographic characteristics of children are presented in Table 1. About 44 to 54% of the study cohorts were Non-Hispanic White Americans followed by about 14 to 16% Mexican Americans. Non-Hispanic Blacks constituted about 12 to 15% of the study cohorts. There were more males than females in all the age segments except in newborn to 5 months old infants. The body mass index (BMI) values appear to be higher as their age increased. We did not statistically analyze the demographic data, as this was not the goal of our current study. This assessment may be of interest in the future.

### 3.2. Age Group 0–0.5 Year (n = 91)

There were very few nutrients for which children at this age range consumed less than the dietary reference intakes (DRIs) (Table 2). Nutrient intake gaps were noted for linolenic acid (LNA), vitamin D, choline, and selenium based on the adequate intake (AI). About one out of three infants consumed LNA and choline, and two out of three infants consumed vitamin D in less than recommended amount. At least two out of five infants consumed less than the AI for selenium. For linoleic acid (LA), about one out of six infants had a nutrient intake gap. There were zero infants with nutrient intake gaps for vitamin B12, pyridoxine, and iron. Most younger infants met the requirement for most nutrients because feeding human milk or infant formulas fulfilled the nutrient needs for this age group. The mean intakes of arachidonic acid (ARA) and docosahexaenoic acid (DHA) were 200 mg and 100 mg, respectively.

### 3.3. Age Group 0.6–1 Year (n = 122)

There were very few nutrients for which 0.6 to 1 year of age consumed less than the DRIs (Table 2). Nutrient intake gaps based on the AI or EAR included LNA, vitamin A, vitamin D, choline, iron, and magnesium. About one out of five older infants had inadequate intake of LA and vitamin E, and about one out of four older infants had an inadequate intake of vitamin A, iron, and magnesium. Approximately two out of five older infants had an intake gap for choline. For vitamin D, three out of five infants consumed less than the recommended amount. At least, one out of six infants did not meet the recommended level for LA. The mean intake of ARA was 200 mg and that for DHA was 100 mg.

### 3.4. Age Group 1–4 Years (n = 713)

The key nutrients consumed at sub-optimal levels in this age segment were dietary fiber, LNA, LA, vitamin A, vitamin D, vitamin E, vitamin K, choline, calcium, and potassium relative to their AI levels (Table 3). However, only vitamin D, vitamin E, and calcium intake were consumed in less than the recommended EAR levels by greater than 20% of the children in this age group. At least 9 out of 10 children had intake gaps for dietary fiber and potassium (AI); about 1 out of 2 children had intake gaps for vitamin K relative to its AI; and 3 out of 5 children consumed less than the AI for LNA, LA, calcium, and iron. Four out of five children and three out of five children did not meet the EAR for vitamin D and E, respectively. About one out of six children in this age group did not consume the EAR for vitamin C. There were no infants with an intake gap for vitamin K in this age group. The mean intakes of ARA and DHA were 100 mg and 0 mg, respectively.

### 3.5. Age Group 5–8 Years (n = 767)

Dietary fiber, LNA, LA, vitamin A, vitamin C, vitamin E, folate, choline, iron, calcium, and potassium were the nutrients with intake gaps in this age segment (Table 3). Nearly all subjects were not meeting the AI for dietary fiber and potassium. About four out of five and one out two children did not consume adequate amount of vitamin D and vitamin E, respectively. Furthermore, about two out of three children did not receive the AI for vitamin K and choline. For calcium and vitamin C, two out of five and one out of four children are not meeting the EAR, respectively. Nearly one out of five children did not meet the EAR for vitamin A. The mean intakes of ARA and DHA were 100 mg and 0 mg, respectively.

### 3.6. Age Group 9–14 Years (n = 996)

The nutrient intake at risk for this age group included dietary fiber, LNA, LA, vitamin A, vitamin C, vitamin D, vitamin E, vitamin K, pyridoxine, folate, choline, calcium, magnesium, zinc, copper, and phosphorus (Table 4). Nearly all children in this age segment had dietary fiber and potassium intake that was less than the recommended AI. Nutrient intake gaps occurred in about two out of five children for LNA, LA, vitamin A, and phosphorus; in nearly one in two children for vitamin C and magnesium; in one out of three children for folate and calcium; and in one out of four children for zinc. Based on recommended AI levels, three out of five and four out of five children in this age group consumed less than the AI levels for vitamin K and choline, respectively. In contrast, intake gaps for pyridoxine and copper were not as large but were in about one out of six children for pyridoxine and in nearly one out of five children for copper. The mean intakes of ARA and DHA in this age group were 100 mg and 0 mg, respectively.

#### Gender Differences in 9–14 Years of Age Group

**Figure 1:**
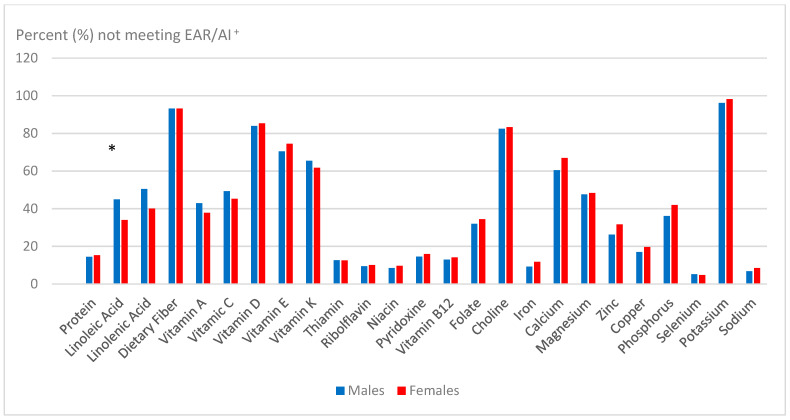
Nutrient intake gaps among US male and female children 9–14 years of age group in 2015–2016 NHANES. + Bar with * denotes significant gender difference at *p* < 0.05.

Mean intakes for all nutrients in this age group were numerically higher for males versus females, with many of them being significantly (*p* < 0.05) different (Table 5). Children with nutrient intake gaps in this age group were numerically (but not statistically, *p* > 0.05) higher in female vs. males for many of the nutrients, including vitamin D, vitamin E, riboflavin, niacin, pyridoxine, vitamin B6, vitamin B12, folate, iron, calcium, zinc, and phosphorus (Table 4 and Figure 1). However, significantly more male children had intake gap for LA (*p* < 0.05) compared with female children.

### 3.7. Age Group 15–19 Years (n = 712)

For this oldest age group in our study, at least one in six children had intake at less than the recommended AI levels for all nutrients except for selenium and sodium (Table 4). For vitamin E and magnesium, four in five children did not consume EAR levels. At least three out of five children had vitamin A and vitamin C, and two out of three children had calcium intake at levels lower than the EAR, respectively. About half (~50%; one in two) of the children consumed LNA, LA, and folate at less than recommended levels. Dietary fiber, vitamin D, vitamin C, choline, and potassium were the top nutrients of concern, with over 90% of children having nutrient intake gaps. For zinc, two in five children; for protein, phosphorus, and copper, one in three children; for thiamin, pyrimidine, and vitamin B12, one in four children; and for iron and riboflavin, one in five children did not receive the recommended nutrient levels, respectively. The mean intakes of ARA and DHA in this age group were 100 mg and 0 mg, respectively.

#### Gender Differences in 15–19 Years of Age Group

**Figure 2:**
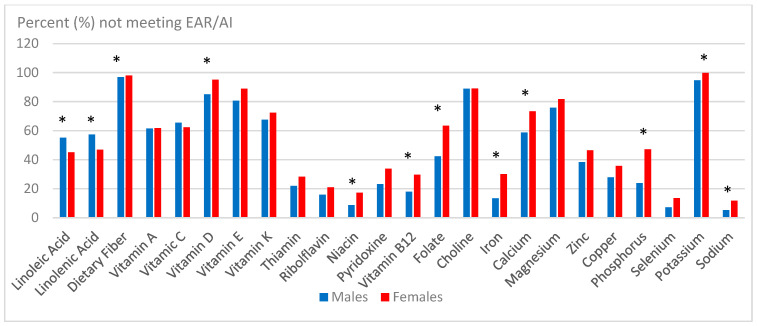
Nutrient intake gaps among US male and female children 15–19 years of age group in 2015–2016 NHANES. Bar with * denotes significant gender difference at *p* < 0.05.

For this oldest age group, the mean intake values for all nutrients were numerically higher for males versus females with most of them being significantly (*p* < 0.05) different (Table 6). There was also gender-specific differences in nutrient intake gaps in this age group (Table 6 and Figure 2). Females had higher nutrient intake gaps (*p <* 0.05) for vitamin D, thiamin, riboflavin, vitamin B12, folate, iron, calcium, folate, magnesium, phosphorus, and potassium compared to males. In contrast, males had significantly (*p* < 0.05) greater intake gaps than females for LA.

### 3.8. Impact of Age on Nutrient Intake Deficits

**Figure 3:**
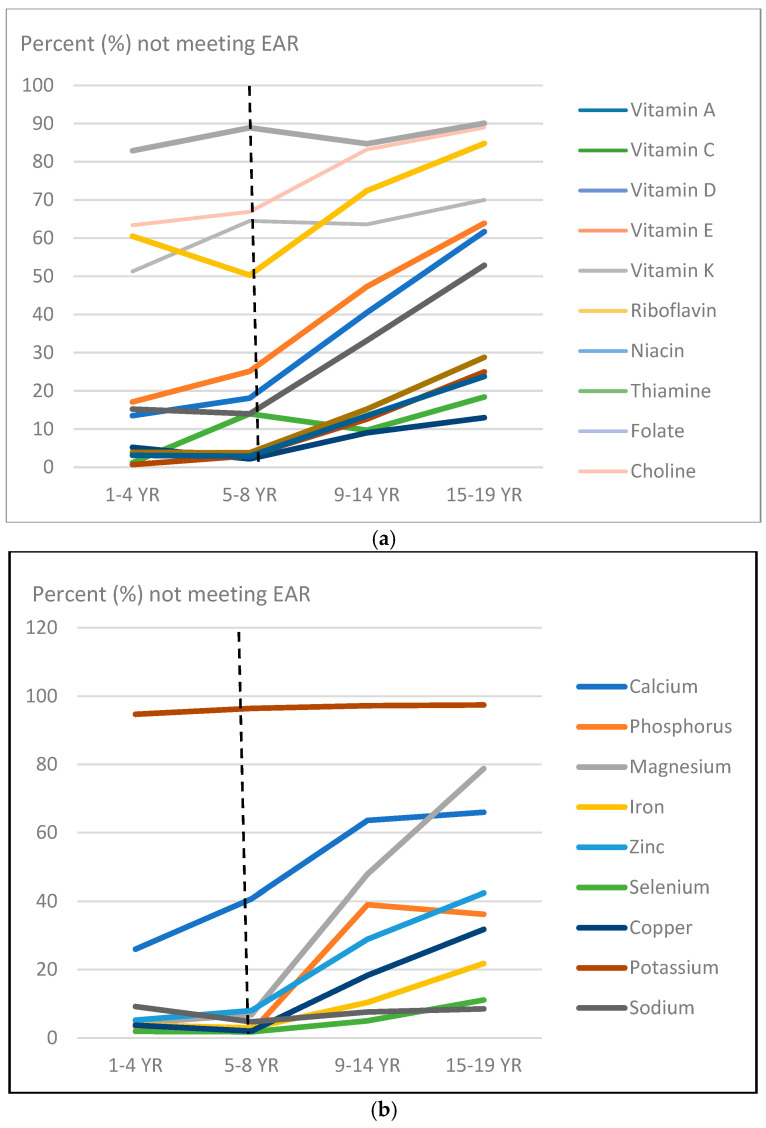
The proportion of subjects in age groups over one year of age not meeting the EAR for some nutrients. The dotted line represents the nutritional intake deficit point of inflection, the age at which nutrient intake gaps drastically increase. (**a**) Intake gaps for vitamins among children in the 2015–2016 NHANES cohort. (**b**) Intake gaps for minerals among children in the 2015–2016 NHANES cohort.

The impact of age on some identified nutrients with nutrient deficits/gaps at both time points is shown in Figure 3. There were fewer numbers of children with nutrient intake gaps at the younger ages compared to the older ages. The numbers of children with key nutrients intake gaps increased dramatically during school ages with the point of inflection at 5–8 years of age. (Figure 3).

### 3.9. Biochemical Correlations

Correlations between log transformed laboratory values available in NHANES 2015–2016 database and their respective nutrient intakes are shown in Table 7. Their intake of iron was significantly associated with laboratory values of hemoglobin (*p* < 0.001) and hematocrit (*p* < 0.001) but not with total iron binding capacity and blood levels of ferritin (all *p* > 0.05). Significant positive associations were noted between folate intake and the blood level of folate (*p* < 0.0126) and red blood cell level of folate (*p* = 0.0458); vitamin D intake and blood levels of vitamin D (*p* < 0.0001); copper intake and the blood levels of copper; and protein intake and the blood level of albumin (*p* < 0.0337) and blood level of urinary nitrogen (*p* < 0.0001) but not with blood level of protein. These positive associations provide some support in validating the nutritional intake data collected from the NHANES.

## 4. Discussion

In our current study, there were fewer nutrient intake gaps identified for infants 0 to 5 months and 6 to 12 months of age compared to any other age evaluated. This finding is consistent with the fact that infants in the US, in most instances, are well nourished by breast milk and/or infant formulas (IFs). Infant feeding recommendations in the US [17] include breastfeeding or formula feeding (when breastfeeding is not chosen or possible) throughout the first year of life with the introduction of solid food at about 4–6 months of age. The DRIs for infants 12 months of age and younger are based on the composition of human milk (HM). The consumption of either HM or IF with appropriate introduction of solid foods has contributed to fewer infants with nutrient intake gaps noted for these age groups. The feeding of breast milk or infant formula progressively diminishes with age during the second half of infancy with replacement with solid foods [18]. Characteristically, solid foods are poor in linoleic acid (LA) and iron [19,20,21].

For children who are 1 year and above, the DRIs are the major factor driving the trends and sizes of the identified nutrient gaps. For example, the recommendations for dietary fiber intake in children have increased over time, as more evidence is available to support the role of dietary fiber in gastrointestinal and metabolic health [22]. However, at least 95% of children 1 year old and above evaluated in our current study have dietary fiber intake gaps. Dietary fiber intake is below recommended amounts for individuals of all ages, not just younger children. The intake of LNA and LA, the two essential fatty acids (EFAs), which cannot be synthesized endogenously, also remained below recommended intakes across all age groups. EFAs are important in cognitive function for both children and adults; thus, if intake is low during critical cognitive development years, the child may have lifelong deficits. Many children in the US are not meeting EFA requirements and, instead, are consuming greater amounts of saturated fat [23]. Unlike LA and LNA, which had DRIs (AIs), docosahexaenoic acid (DHA) and arachidonic acid (ARA) did not have DRIs in the US. Based on the proposal by the European Food Safety Authority, EFSA [24] regarding AI for DHA at 100 mg per day for infants 7 to 12 months of age and 250 mg per day for combination of DHA and EPA (eicosatetraenoic acid) for 2 years of age and above, it appears that the intake of DHA in the US is near adequate for infants and toddlers. This is consistent with the fact that human milk and most infant formulas in the US contain adequate amounts of DHA and ARA. In contrast, it is not surprising that the intake of DHA and ARA are low in school-age children and older children in the US given the low intake of sea foods, which is as a source of DHA, in these age groups.

In addition, we found nutrient intake gaps for vitamins C, E, and D and calcium in children 1 year and above in our current study, which are consistent with the findings in other studies. Vitamins E and D and calcium nutrient intakes were reported to be significantly below the EAR in 1- to 6-year-old children in NHANES 2001–2016 [25]. Iron, zinc, vitamin C, and essential fatty acids were the nutrients commonly found to be taken in less than recommended levels in toddlers and older infants in the US [19,20,21]. These were also the nutrients found to be low in cow’s milk, the major beverage consumed by toddlers in the US. Efforts to fortify cow’s milk with some of these key nutrients could enhance the nutritional status of toddlers in the US.

Though certain nutrient intake gaps were common in all age groups, we found more nutrient intake gaps among the older age groups (9–14- and 15–19-year age groups). This may be due in part to the fact that as children grow older and become more independent, they consume more of their food outside the home and can make their own choices, independent of their parents. Studies [26,27] have noted significant changes in the consumption pattern of major food groups in children, which can have a significant impact on their nutrient intakes. Of these pattern changes, the beverage consumption trend in school-aged children was the most important. In general, there is a notable decrease in the consumption of milk and dairy products, beans, vegetables, and whole fruits, with a significant increase in intakes of carbonated soft drinks, non-citrus fruit drinks/juices, and snacks such as corn chips, popcorns, pretzels, and crackers in school children. The resultant effects are the prevalence of a sub-adequate intake of calcium, phosphorus, magnesium, vitamins A and C, and folate, which are abundant in milk, fruits, beans, and vegetables [26,27]. These nutrient intake patterns could predispose children to a greater risk for chronic diseases such as osteoporosis, type 2 diabetes, heart disease, and hypertension in later life. The excessive consumption of carbonated soft drinks among adolescent females was associated with bone fractures [28], with implications for slow bone growth and higher risks for osteoporosis and obesity in later life.

The identification of gender-specific nutrient intake gaps was possible in children 9 years of age and older because the DRIs are differentiated for some nutrients at this age group. In this current study, the most and largest nutrient intake gaps were noted in the children who are 15–19 years of age and especially in females. Thus, the older the children are, the more nutrient intake gaps they have. The bigger nutrient intake gaps noted with female adolescents compared with males for iron, zinc, and folate was to be expected, as females mature earlier than boys and have higher requirements for these nutrients in order to compensate for losses during menstrual periods. Other studies [26,27] have also reported a higher prevalence of inadequate intakes for many essential nutrients in school-aged females compared with males. The highest prevalence of inadequate nutrient intake among children was reported [29] to be in females 14–18 years of age for vitamins A, C, E, and B_6_ and folate as well as for phosphorus, calcium, iron, and zinc. These are consistent with some of the nutrient gaps noted in our current study. It is possible that there is a greater need for nutrition education and dietary intervention for females than males to ensure that nutrient requirements are met as children continue to grow. Poor nutritional status can impact pubertal and cognitive development and may affect later fertility among women [26].

Overall, there is a large distinction between the nutrient intake patterns of non-school-aged children compared to school-aged children. This is reflective of several factors, including the progressive loss of parental ability to control what their children consume at school compared to home. The age ranges for this change in food control or direction appeared to be 9–14 and 15–19 years of age. These age segments need a vigorous nutrition intervention program.

It is well known that dietary intake surveys are often subjective, with underestimation and overestimation of some specific nutrients [30,31]. To verify the reliability of the intake data in our current study, we assessed the correlation of available laboratory biomarkers with nutrient intake in the NHANES 2015–2016 database. It is encouraging that we were able to obtain validated nutrient intake for vitamin D, folate, iron, copper, and protein using laboratory blood biomarkers.

The strength of our study included providing a comprehensive review of key nutrient intakes at risk in a meaningful segmentation into different age groups of children. The study is particularly unique in identifying the age group of inflection (5–8 years of age; school age) for the US children at which the numbers of nutrients with intake gaps increased exponentially. This could provide special focus and intervention directed at this age group to aid transition from meals to school lunches. In addition, the study identified gender-specific differences in key nutrient intake gaps that might benefit targeted gender nutritional intervention, nutritional innovation, or emerging personalized nutrition. A good example is proving the female teenagers/athletes with iron-, magnesium-, folate-, and calcium-rich foods. The limitations of this study include the fact that we did not have more comprehensive laboratory markers to correlate with more nutrient intakes in the NHANES 2015–2016 database, unlike the NHANES 2013–2014 database, which had a very elaborate number of laboratory biomarkers to validate nutrient intakes. Our future study will improve on this by using the most currently available and validated NHANES database. Lastly, the limitation of this study is the subjectivity of survey studies, which rely on recalls. Future studies are needed to improve on the reliability and validation of cross-sectional survey studies.

## 5. Conclusions

In summary, this study evaluated nutrient intake data among children from a major national nutrition survey in the US and identified key essential nutrients consumed in inadequate amounts among children. The identified nutrient intake gaps were for fiber, vitamin A, vitamin D, vitamin E, vitamin K, folate, choline, essential fatty acids (linoleic acid, LA, and linolenic acid, LNA), docosahexaenoic acid (DHA), calcium, and iron in most age segments of US children. Implications may be made regarding the possible use of these identified nutrient gaps for planning and implementation of nutritional interventions. Furthermore, the identified nutrient intake gaps might provide potential opportunities to develop targeted key nutrient-rich-specific products for different ages and genders in the US population of children.

## Figures and Tables

**Table 1:** Subjects’ demographics *.

Measures	0–5 Months of Age (*n* = 91)	0.5–1 Year of Age (*n* = 122)	1–4 Years of Age (*n* = 713)	5–8 Years of Age (*n* = 676)	9–14 Years of Age (*n* = 996)	15–19 Years of Age (*n* = 712)
Numbers of subjects, *n*; (Percent ± SEM Female/Percent ± SE for Males)	51 (55.09 ± 6.35)/ 40 (44.91 ± 6.35)	62 (45.26 ± 4.76)/ 60 (54.74 ± 4.76)	349 (49.87 ± 1.76)/ 364 (50.13 ± 1.76)	327 (46.15 ± 2.81)/ 349 (53.85 ± 2.81)	497 (48.83 ± 1.62)/ 499 (51.17 ± 1.62)	355 (49.93%, 1.84)/ 357 (50.07%, 1.84)
Mean Age at Screening (years)	0.23 ± 0.14 (91)	0.71 ± 0.18 (122)	2.55 ± 0.03 (713)	6.59 ± 0.06 (676)	11.68 ± 0.06 (996)	16.72 ± 0.05 (712)
Weight (kg)	6.70 ± 0.12 (91)	9.20 ± 0.13 (122)	15.12 ± 0.17 (703)	26.30 ± 0.45 (673)	50.78 ± 0.97 (991)	71.31 ± 0.80 (703)
Height (cm)	Not measured	Not measured	99.02 ± 0.44 (490)	122.38 ± 0.51 (673)	152.82 ± 0.53 (990)	168.16 ± 0.45 (703)
BMI (kg/m^2^)	Not available	Not available	16.52 ± 0.09 (488)	17.26 ± 0.18 (673)	21.27 ± 0.32 (990)	25.18 ± 0.30 (703)
Ethnicity: *n* (Percent, SEM)						
Mexican American	27 (21.91%, 7.57)	24 (13.50%, 3.92)	135 (15.41%, 3.31)	152 (16.25%, 4.06)	216 (14.25%, 3.93)	163 (15.45%, 3.52)
Other Hispanic	13 (10.02%, 3.83)	20 (10.46%, 2.65)	81 (8.82%, 1.60)	93 (9.42%, 1.54)	135 (9.58%, 1.35)	72 (6.63%, 1.19)
Non-Hispanic White	25 (44.35%, 9.40)	38 (46.40%, 7.11)	238 (53.70%, 5.50)	189 (51.56%, 6.28)	282 (52.84%, 5.96)	193 (53.85%, 5.33)
Non-Hispanic Black	16 (12.26%, 4.35)	25 (15.06%, 5.12)	160 (12.36%, 2.98)	155 (13.76%, 3.36)	219 (13.72%, 3.39)	163(14.07%, 3.16)
Others including multi-racial	10 (11.47%, 3.53)	15 (14.37%, 3.51)	99 (9.71%, 1.80)	87 (9.01%, 1.17)	144 (9.60%, 1.86)	121 (9.99%, 1.61)

* Subjects with Mobile Equipment Center (MEC) Examination and Dietary Data and were, not pregnant.

**Table 2:** Selected nutrient intake at risk among US children in the 2015–2016 NHANES cohorts: 0.0–0.5, and 0.5–1 year of age group.

	0.0–0.5 Year of Age Group (*n* = 91)			0.5–1 Year of Age (*n* = 122)		
Nutrients	Intake/Day	% Intake at Risk (AI)	% Intake at Risk (EAR)	Intake/Day	% Intake at Risk (AI)	% Intake at Risk (EAR)
Macronutrients						
Protein (g)	14.1 ± 0.5	7.0 ± 2.5	NA	23.2 ± 1.5	6.2 ± 3.0	1.5 ± 1.3
Linoleic Acid (g)	5.8 ± 0.2	17.3 ± 3.7	NA	6.9 ± 0.4	18.6 ± 5.2	NA
Linolenic Acid (g)	0.6 ± 0.0	34.7 ± 3.9	NA	0.7 ± 0.0	21.5 ± 6.0	NA
Arachidonic Acid (g)	0.2 ± 0.0	NA	NA	0.2 ± 0.0	NA	NA
Docosahexaenoic Acid (g)	0.1 ± 0.0	NA	NA	0.1 ± 0.0	NA	NA
Dietary Fiber (g)	0.5 ± 0.1	NA	NA	5.1 ± 0.4	NA	NA
Vitamins						
A	596.5 ± 29.3	13.9 ± 3.7	NA	752.8 ± 32.7	24.8 ± 5.0	NA
C (mg)	68.8 ± 3.2	4.5 ± 2.4	NA	93.0 ± 4.4	11.7 ± 3.4	NA
D (mcg)	9.6 + 0.3	60.9 ± 6.7	NA	9.3 ± 0.4	61.4 ± 8.2	NA
E (mg)	7.3 ± 0.3	5.9 ± 2.9	NA	7.8 ± 0.4	20.5 ± 4.8	NA
K (mcg)	52.6 ± 1.6	0.0 ± 0.0	NA	65.0 ± 5.9	0.0 ± 0.0	NA
Thiamin (µg)	0.6 ± 0.0	0.5 ± 0.4	NA	0.8 ± 0.0	1.3 ± 1.3	NA
Riboflavin (µg)	0.9 ± 0.0	1.0 ± 0.7	NA	1.2 ± 0.1	1.3 ± 1.3	NA
Niacin (µg)	7.0 ± 0.2	0.5 ± 0.4	NA	10.2 ± 0.5	1.0 ± 0.8	NA
Pyridoxine (mg)	0.4 ± 0.0	0.0 ± 0.0	NA	0.8 ± 0.0	1.0 ± 0.9	NA
B12 (µg)	1.9 ± 0.1	0.0 ± 0.0	NA	2.5 ± 0.1	0.0 ± 0.0	NA
Folate (µg)	103.1 ± 3.8	7.9 ± 2.9	NA	156.3 ± 8.7	6.5 ± 2.4	NA
Choline (mcg)	144.9 ± 4.4	32.0 ± 4.3	NA	174.8 ± 10.5	40.0 ± 8.3	NA
Minerals						
Iron (mg)	12.7 ± 0.5	0.0 ± 0.0	NA	15.9 ± 0.7	27.7 ± 5.0	5.5 ± 1.8
Calcium (mg)	557.4 ± 20.8	1.0 ± 0.7	NA	698.6 ± 32.1	2.4 ± 1.5	NA
Magnesium (mg)	56.0 ± 2.7	7.2 ± 3.8	NA	101.9 ± 5.3	27.9 ± 5.2	NA
Zinc (mg)	5.9 ± 0.3	1.0 ± 0.7	NA	7.4 ± 0.4	2.4 ± 1.5	2.4 ± 1.5
Copper (mg)	0.5 ± 0.0	0.5 ± 0.4	NA	0.7 ± 0.0	1.6 ± 1.3	NA
Phosphorus (mg)	321.8 ± 17.0	0.6 ± 0.6	NA	509.3 ± 27.2	13.7 ± 6.1	NA
Selenium (µg)	16.0 ± 0.8	45.6 ± 8.1	NA	30.5 ± 3.2	41.1 ± 5.7	NA
Potassium (mg)	722.5 ± 28.8	1.5 ± 0.8	NA	1192.2 ± 47.1	6.1 ± 2.8	NA
Sodium (mg)	211.9 + 10.8	8.0 ± 3.0	NA	576.2 ± 58.7	57.0 ± 3.4	NA

Intake is presented as mean ± standard error of the mean (SEM). NHANES = National Health and Nutrition Examination Survey; AI = adequate intake; EAR = estimate average requirement; NA = Not assessed because EAR was not defined.

**Table 3:** Selected nutrient intake at risk among US children in the 2015–2016 NHANES cohorts: 1–4 and 5–8 years of age groups.

	1–4 Years of Age Group (*n* = 713)			5–8 Years of Age Group (*n* = 676)		
Nutrients	Intake/Day	% Intake at Risk (AI)	% Intake at Risk (EAR)	Intake/Day	% Intake at Risk (AI)	% Intake at Risk (EAR)
Macronutrients						
Protein (g)	50.0 ± 0.9	0.6 ± 0.3	0.4 ± 0.2	61.7 ± 1.1	1.2 ± 0.5	2.0 ± 0.7
Linoleic Acid (g)	9.6 ± 0.0	46.2 ± 2.9	NA	13.3 ± 0.3	35.8 ± 1.7	NA
Linolenic Acid (g)	1.0 ± 0.0	40.1 ± 2.9	NA	1.2 ± 0.0	38.3 ± 2.6	NA
Arachidonic Acid (g)	0.1 ± 0.0	NA	NA	0.1 ± 0.0	NA	NA
Docosahexaenoic Acid (g)	0.0 ± 0.0	NA	NA	0.0 ± 0.0	NA	NA
Dietary Fiber (g)	10.7 ± 0.2	93.8 ±1.0	NA	13.3 ± 0.4	95.5 ± 0.8	NA
Vitamins						
A	528.2 ± 15.2	28.6 ± 1.8	13.5 ± 2.1	608.0 ± 30.6	33.0 ± 2.4	18.1 ± 2.0
C (mg)	68.8 ± 4.4	18.7 ± 2.7	17.1 ± 2.8	65.7 ± 5.5	29.3 ± 4.5	25.1 ± 4.0
D (mcg)	6.2 + 0.2	95.6 ± 1.0	82.9 ± 1.9	5.6 ± 0.2	98.1 ± 0.5	88.9 + 1.6
E (mg)	5.3 ± 0.1	72.0 ± 2.0	60.5 ± 2.8	6.7 ± 0.1	60.5 ± 2.3	50.3 ± 1.9
K (mcg)	47.4 ± 2.7	51.3 ± 2.7	NA	58.2 ± 3.3	64.5 ± 2.5	NA
Thiamin (µg)	1.1 ± 0.0	6.2 ± 1.0	3.5 ± 0.9	1.4 ± 0.0	4.9 ± 1.1	3.0 ± 0.9
Riboflavin (µg)	1.6 ± 0.0	1.5 ± 0.5	0.7 ± 0.3	1.9 ± 0.0	2.5 ± 0.5	1.7 ± 0.5
Niacin (µg)	14.4 ± 0.4	9.5 ± 1.5	5.2 ± 1.0	19.0 ± 0.6	6.8 ± 1.3	2.2 ± 0.6
Pyridoxine (mg)	1.3 ± 0.0	8.3 ± 1.7	3.9 ± 0.8	1.5 ± 0.1	5.9 ± 1.0	3.8 ± 0.9
B12 (µg)	1.9 ± 0.1	5.1 ± 1.3	3.1 ± 0.8	1.9 ± 0.0	4.4 ± 0.7	2.9 ± 0.7
Folate (µg)	268.5 ± 6.0	27.1 ± 2.2	15.2 ± 1.3	337.3 ± 9.6	22.3 ± 2.7	14.0 ± 2.8
Choline (mg)	205.4 ± 5.7	63.4 ± 2.5	NA	227.7 ± 6.4	66.9 ± 2.9	NA
Minerals						
Iron (mg)	9.9 ± 0.2	40.5 ± 1.9	3.8 ± 0.9	12.9 ± 0.4	37.3 ± 2.0	2.9 ± 0.8
Calcium (mg)	913.7 ± 15.1	43.7 ± 1.5	26.0 + 1.5	971.2 ± 26.5	59.5 ± 2.6	40.7 ± 3.1
Magnesium (mg)	183.7 ± 3.0	9.3 ± 1.7	4.2 ± 1.2	218.0 ± 4.1	11.9 ± 1.7	6.8 ± 1.3
Zinc (mg)	7.3 ± 0.1	10.5 ± 1.3	3.7 ± 0.6	9.4 ± 0.2	13.0 ± 1.9	8.0 ± 1.4
Copper (mg)	0.7 ± 0.0	11.4 ± 1.7	3.7 ± 0.7	0.9 ± 0.0	7.6 ± 1.3	2.0 ± 0.6
Phosphorus (mg)	1010.1 ± 17.2	3.5 ± 0.9	1.4 ± 0.5	1187.3 ± 23.3	4.4 ± 0.7	1.8 ± 0.6
Selenium (µg)	70.2.0 ± 1.5	3.2 ± 1.2	1.2 ± 0.4	89.7 ± 1.5	3.5 ± 0.8	1.8 ± 0.7
Potassium (mg)	1812.3 ± 34.1	94.7 ± 0.6	NA	1995.0 ± 56.2	96.4 ± 0.9	NA
Sodium (mg)	2028.5 ± 40.0	9.2 ± 1.5	NA	2736.9 ± 50.9	4.7 ± 0.9	NA

Intake is presented as mean ± standard error of the mean (SEM). AI = adequate intake; EAR = estimate average requirement; NA = Not assessed because the DRI was not defined.

**Table 4:** Selected nutrient intake at risk among US children in the 2015–2016 NHANES cohorts: 9–14 and 15–19 years of age groups (combined males and females).

	9–14 Years of Age Group (*n* = 996)			15–19 Years of Age Group (*n* = 712)		
Nutrients	Intake/Day	% Intake at Risk (AI)	% Intake at Risk (EAR)	Intake/Day	% Intake at Risk (AI)	% Intake at Risk (EAR)
Macronutrients						
Protein (g)	72.8 ± 1.4	12.1 ± 1.7	14.9 + 2.0	74.1 ± 2.9	27.8 ± 3.5	30.1 ± 2.8
Linoleic Acid (g)	15.8 ± 0.3	39.6 ± 1.7	NA	16.1 ± 0.5	50.1 ± 2.3	NA
Linolenic Acid (g)	1.5 ± 0.1	45.4 ± 2.8	NA	1.6 ± 0.1	52.1 ± 2.0	NA
Arachidonic Acid (g)	0.1 ± 0.0	NA	NA	0.1 ± 0.0	NA	NA
Docosahexaenoic Acid (g)	0.0 ± 0.0	NA	NA	0.0 ± 0.0	NA	NA
Dietary Fiber (g)	15.3 ± 0.5	93.2 ± 1.2	NA	14.6 ± 0.4	97.5 ± 0.6	NA
Vitamins						
A (RE)	650.2 ± 32.5	58.9 ± 2.5	40.5 ± 3.2	526.6 ± 26.3	76.4 ± 2.5	61.7 ± 3.4
C (mg)	70.6 ± 4.5	51.5 ± 2.9	47.3 ± 2.5	62.6 ± 3.8	67.4 ± 2.9	63.9 ± 3.4
D (mcg)	5.5 ± 0.3	94.9 ± 0.7	84.7 + 1.7	4.5 ± 0.3	95.3 + 0.8	90.1 + 1.8
E (mcg)	8.0 ± 0.3	83.5 ± 1.5	72.4 ± 1.7	7.6 ± 0.2	92.1 ± 1.5	84.8 ± 1.6
K (mcg)	73.3 ± 4.4	63.6 ± 2.7	NA	75.3 ± 4.3	70.0 ± 3.5	NA
Thiamin (µg)	1.7 ± 0.2	18.2 ± 1.7	12.6 ± 1.7	1.6 ± 0.0	34.0 ± 1.9	25.0 ± 2.4
Riboflavin (µg)	2.0 ± 0.1	13.4 ± 1.4	9.7 ± 1.0	1.9 ± 0.1	29.7 ± 2.2	18.4 ± 2.3
Niacin (µg)	23.4 ± 0.6	16.8 ± 1.6	9.0 ± 1.2	24.4 ± 0.9	24.9 ± 1.6	13.0 ± 1.0
Pyridoxine (mg)	1.8 ± 0.1	23.3 ± 2.2	15.2 ± 1.5	1.8 ± 0.1	37.7 ± 2.7	28.8 ± 2.2
B12 (µg)	4.9 ± 0.2	18.6 ± 1.8	13.5 ± 1.5	4.8 ± 0.3	30.9 ± 3.0	23.8 ± 2.8
Folate (µg)	397.1 ± 12.8	43.3 ± 2.6	33.2 ± 2.5	372.3 ± 12.4	65.7 ± 2.6	52.9 ± 2.5
Choline (mcg)	267.7 ± 7.4	83.2 ± 2.0	NA	268.6 ± 13.3	89.0 ± 1.8	NA
Minerals						
Iron (mg)	15.8 ± 0.5	21.9 ± 1.6	10.4 ± 1.6	14.0 ± 0.4	52.6 ± 2.7	21.8 ± 2.3
Calcium (mg)	1034.6 ± 37.3	74.7 ± 2.5	63.6 ± 2.8	966.9 ± 45.7	74.5 ± 2.8	66.0 ± 2.7
Magnesium (mg)	249.1 ± 7.3	60.7 ± 2.1	47.9 ± 2.4	246.0 ± 5.9	88.4 ± 1.5	78.8 ± 2.7
Zinc (mg)	11.1 ± 0.3	38.9 ± 2.3	28.9 ± 1.8	10.3 ± 0.5	57.7 ± 3.3	42.4 ± 3.1
Copper (mg)	1.0 ± 0.0	36.3 ± 2.3	18.3 + 2.4	1.0 ± 0.0	53.1 ± 2.8	31.8 + 3.0
Phosphorus (mg)	1324.0 ± 35.2	52.1 ± 3.1	39.0 ± 2.3	1300.2 ± 48.0	50.2 ± 2.8	36.2 ± 3.0
Selenium (mg)	104.2 ± 2.1	8.1 ± 1.4	5.0 ± 0.7	109.1 ± 3.9	16.6 ± 2.2	11.1 ± 1.5
Potassium (mg)	2208.9 ± 47.7	97.2 ± 0.7	NA	2166.4 ± 75.1	97.4 ± 0.7	NA
Sodium (mg)	3302.1 ± 67.8	7.6 ± 1.1	NA	3402.4 ± 85.7	8.5 ± 1.4	NA

Intake is presented as mean ± standard error of the mean (SEM). AI = adequate intake; EAR = estimate average requirement; NA = Not assessed because the DRI was not defined.

**Table 5:** Selected nutrient intake at risk among US male and female children 9–14 years of age group in 2015–2016 NHANES.

	Intake/day			% Intake at Risk (AI)		% Intake at Risk (EAR)		
Nutrients	Males (*n* = 499)	Females (*n* = 497)	*p*-Values ^+^	Males (*n* = 499)	Females (*n* = 497)	Males (*n* = 499)	Females (*n* = 497)	*p*-Values (AI or EAR) ^+^
Macronutrients								
Protein (g)	76.9 ± 2.4	68.4 ± 1.7	0.0130 *	12.1 ± 2.3	12.1 ± 2.0	14.4 ± 2.7	15.3 ± 2.6	0.7900
Linoleic Acid (g)	16.0 ± 0.6	15.1 ± 0.5	0.3680	45.0 ± 3.0	34.0 ± 3.0	NA	NA	0.0450 *
Linolenic Acid (g)	1.5 ± 0.1	1.5 ± 0.1	0.6410	50.5 ± 3.2	40.1 ± 4.4	NA	NA	0.0570
Arachidonic Acid (g)	0.1 ± 0.0	0.1 ± 0.0	0.0300 *	NA	NA	NA	NA	NA
Docosahexaenoic Acid (g)	0.0 ± 0.0	0.0 ± 0.0	0.7220	NA	NA	NA	NA	NA
Dietary Fiber (g)	15.5 ± 0.6	15.1 ± 0.5	0.5000	93.2 ± 1.8	93.2 ± 1.7	NA	NA	0.9840
Vitamins								
A (RE)	690.3 ± 49.3	608.5 ± 26.5	0.0860	58.1 ± 3.4	59.7 ± 2.6	43.0 ± 3.3	37.8 ± 3.7	0.0840
C (mg)	74.3 ± 6.5	66.8 ± 3.4	0.1810	53.4 ± 3.5	49.5 ± 3.1	49.3 ± 3.1	45.3 ± 2.6	0.1760
D (mcg)	5.8 ± 0.4	5.3 ± 0.3	0.1400	93.3 ± 1.4	96.6 ± 1.3	84.0 ± 2.4	85.3 ± 2.6	0.7330
E (mcg)	8.4 ± 0.6	7.6 ± 0.3	0.2590	81.8 ± 2.5	85.2 ± 2.2	70.5 ± 3.1	74.5 ± 2.6	0.4160
K (mcg)	74.4 ± 5.7	72.2 ± 7.2	0.8210	65.5 ± 3.0	61.7 ± 4.1	NA	NA	0.4210
Thiamin (µg)	1.8 ± 0.1	1.6 ± 0.0	0.0150 *	17.3 ± 1.9	19.2 ± 2.5	12.6 ± 2.1	12.5 ± 2.6	0.9670
Riboflavin (µg)	2.2 ± 0.1	1.9 ± 0.1	0.0010 *	11.5 ± 2.0	15.3 ± 2.3	9.4 ±1.7	10.1 ± 2.0	0.8160
Niacin (µg)	24.6 ± 0.9	22.0 ± 0.5	0.0090 *	16.1 ± 2.7	17.6 ± 2.1	8.5 ± 1.9	9.6 ± 1.8	0.7260
Pyridoxine (mg)	2.0 ± 0.1	1.7 ± 0.0	0.0160 *	21.7 ± 2.5	24.9 ± 2.8	14.5 ± 2.4	15.9 ± 1.8	0.6640
B12 (µg)	5.3 ± 0.3	4.5 ± 0.1	0.0080 *	16.6 ± 1.7	20.7 ± 2.2	12.9 ± 1.7	14.1 ± 1.9	0.5650
Folate (µg)	173.8 ± 7.8	163 ± 6.7	0.3030	42.3 ± 3.1	44.4 ± 3.9	32.0 ± 3.5	34.4 ± 3.0	0.5800
Choline (mcg)	279.9 ± 10.4	255.0 ± 8.0	0.0400 *	82.5 ± 3.0	83.8 ± 1.8	NA	NA	0.6540
Minerals								
Iron (mg)	16.3 ± 0.7	15.3 ± 0.6	0.1520 *	19.6 ± 1.9	24.3 ± 2.4	9.2 ± 1.7	11.8 ± 2.3	0.2800
Calcium (mg)	1101.6 ± 50.0	964.9 ± 38.1	0.0110 *	71.2 ± 4.1	78.3 ± 2.8	60.4 ± 3.5	66.9 + 3.1	0.0610
Magnesium (mg)	262.4 ± 11.8	235.3 ± 7.0	0.0500 *	57.8 ± 3.7	63.7 ± 3.2	47.6 ± 3.6	48.3 ± 3.4	0.8940
Zn (mg)	11.8 ± 0.4	10.3 ± 0.3	0.0020 *	34.7 ± 2.1	43.4 ± 3.4	26.3 ± 2.0	31.7 ± 3.0	0.1390
Copper (mg)	1.0 ± 0.0	0.9 ± 0.0	0.0950	37.1 ± 3.9	35.4 ± 3.1	17.0 ± 3.1	19.7 ± 2.9	0.4620
Phosphorus (mg)	1391.6 ± 55.3	1253.5 ± 32.4	0.0260 *	47.3 ± 4.3	57.0 ± 3.0	36.1 ± 3.0	42.0 ± 3.2	0.1750
Selenium (mg)	109.3 ± 3.6	98.7 ± 2.8	0.0450 *	7.3 ± 1.8	9.0 ±1.8	5.2 ± 1.2	4.8 ± 0.8	0.7870
Potassium (mg)	2300.4 ± 72.9	2113.5 ± 53.9	0.0420 *	96.2 ± 1.1	98.2 ± 0.9	NA	NA	0.1410
Sodium (mg)	3497.4 ± 131.9	3098.9 ± 79.4	0.0340 *	6.8 ± 1.7	8.5 ± 1.6	NA	NA	0.4920

Intake is presented as mean ± standard error of the mean (SEM). AI = adequate intake; EAR = estimate average requirement; NA = Not assessed because the DRI was not defined. ^+^ *p*-value with * denotes significant gender difference at *p* < 0.05.

**Table 6:** Selected nutrient intake at risk among US male and female children 15–19 years of age group in 2015–2016 NHANES.

	Intake/day			% Intake at Risk (AI)		% Intake at Risk (EAR)		
Nutrients	Males (*n* = 357)	Females (*n* = 355)	*p*-Values ^+^	Males (*n* = 357)	Females (*n* = 355)	Males (*n* = 357)	Females (*n* = 355)	*p*-Values (AI or EAR) ^+^
Macronutrients								
Protein (g)	87.1 ± 4.0	61.1 ± 2.0	0.0001 *	25.3 ± 3.7	30.2 ± 4.3	28.6 ± 4.2	31.6 ± 3.6	0.6130
Linoleic Acid (g)	17.9 ± 0.7	14.3 ± 0.5	0.0020 *	55.2 ± 3.8	45.1 ± 2.6	NA	NA	0.0410 *
Linolenic Acid (g)	1.7 ± 0.1	1.4 ± 0.1	0.0100 *	57.4 ± 3.8	46.9 ± 3.0	NA	NA	0.0710
Arachidonic Acid (g)	0.2 ± 0.0	0.1 ± 0.0	0.0001 *	NA	NA	NA	NA	NA
Docosahexaenoic Acid (g)	0.0 ± 0.0	0.0 ± 0.0	0.1740	NA	NA	NA	NA	NA
Dietary Fiber (g)	16.0 ± 0.6	13.1 ± 0.4	0.0010 *	96.9 ± 0.8	98.1 ± 1.0	NA	NA	0.3430
Vitamins								
A (RE)	593.8 ± 31.0	459.9 ± 27.0	<0.0001 *	76.6 ± 2.7	76.1 ± 3.8	61.5 ± 3.4	61.8 ± 4.8	0.9580
C (mg)	62.8 ± 5.0	62.3 ± 4.4	0.9310	69.9 ± 3.4	64.9 ± 3.8	65.5 ± 3.7	62.3 ± 4.3	0.4780
D (mcg)	5.4 ± 0.5	3.7 ± 0.3	0.0010 *	92.4 ± 1.2	98.1 ± 0.9	85.1 ± 2.3	95.1 + 1.6	<0.0001 *
E (mcg)	8.3 ± 0.4	7.0 ± 0.4	0.0610	90.4 ± 2.0	98.1 ± 0.9	80.7 ± 2.8	88.9 ± 2.6	0.0800 *
K (mcg)	78.3 ± 5.3	72.4 ± 7.3	0.5440	67.6 ± 3.5	72.4 ± 4.4	NA	NA	0.2380
Thiamin (µg)	1.8 ± 0.1	1.4 ± 0.1	<0.0001 *	31.0 ± 3.1	37.1 ± 2.4	21.9 ± 3.3	28.2 ± 2.4	0.0720
Riboflavin (µg)	2.3 ± 0.1	1.6 ± 0.1	<0.0001 *	28.5 ± 2.2	31.0 ± 3.8	15.8 ± 3.5	21.0 ± 2.7	0.2330
Niacin (µg)	28.9 ± 1.1	20.0 ± 0.7	<0.0001 *	20.7 ± 3.1	29.0 ± 3.1	8.7 ± 2.4	17.2 ± 1.9	0.0470 *
Pyridoxine (mg)	2.1 ± 0.1	1.6 ± 0.1	<0.0001 *	31.1 ± 3.2	44.3 ± 4.5	23.8 ± 3.1	33.8 ±3.2	0.0580
B12 (µg)	6.0 ± 0.4	3.6 ± 0.2	<0.0001 *	24.4 ± 3.0	37.4 ± 4.8	17.9 ± 3.5	29.7 ± 3.9	0.0340 *
Folate (µg)	189.5 ± 6.9	145.5 ± 4.4	<0.0001 *	55.0 ± 3.0	76.3 ± 3.8	42.3 ± 2.9	63.4 ± 2.9	<0.0001 *
Choline (mcg)	315.1 ± 19.5	222.5 ± 11.4	<0.0001 *	88.9 ± 2.0	89.1 ± 3.0	NA	NA	0.9460
Minerals								
Iron (mg)	16.2 ± 0.7	11.9 ± 0.4	0.1520	29.0 ± 3.2	75.9 ± 2.5	13.4 ± 2.6	30.1 ± 3.0	<0.0001 *
Calcium (mg)	1103.7 ± 57.6	831.2 ± 48.2	<0.0001 *	66.5 ± 3.6	82.5 ± 3.5	58.7 ± 3.0	73.3 ± 3.3	<0.0001 *
Magnesium (mg)	273.9 ± 9.8	218.8 ± 6.1	<0.0001 *	84.6 ± 2.1	92.2 ± 1.6	75.9 ± 2.3	81.7 ± 2.3	0.1150
Zn (mg)	12.1 ± 0.7	8.6 ± 0.4	<0.0001 *	53.7 + 4.5	61.8 ± 3.3	38.3 ± 3.9	46.4 ± 3.5	0.0690
Copper (mg)	1.1 ± 0.0	0.9 ± 0.0	<0.0001 *	44.0 ± 3.9	62.2 ± 2.9	27.9 ± 4.1	35.7 ± 3.5	0.1190
Phosphorus (mg)	1491.5 ± 62.9	1110.3 ± 44.1	<0.0001 *	39.9 ± 3.3	60.5 ± 3.5	25.3 ± 3.5	47.1 ± 3.5	<0.0001 *
Selenium (mg)	128.5 ± 6.5	89.9 ± 2.4	<0.0001 *	12.3 ± 2.3	20.7 ± 2.8	8.7 ± 2.0	13.5 ± 2.0	0.0790
Potassium (mg)	2437.1 ± 112.2	1898.0 ± 56.3	<0.0001 *	94.8 ± 1.3	99.9 ± 0.1	NA	NA	0.0020 *
Sodium (mg)	3913.6 ± 116.7	2895.4 ± 79.6	<0.0001 *	5.2 ± 1.4	11.7 ± 2.0	NA	NA	0.0070 *

Intake is presented as mean ± standard error of the mean (SEM). AI = adequate intake; EAR = estimate average requirement; NA = Not determined because the DRI was not defined. ^+^ *p*-value with * denotes significant gender difference at *p* < 0.05.

**Table 7:** Associations between selected nutrient intakes and log transformed laboratory blood values in the NHANES 2015–2016 children.

Independent Variables (Nutrient Intakes)	Dependent Variables (Blood Levels)	Beta Coefficient	Standard Error	R Square	*p* Value
Vitamin D (mcg)	Log of Vitamin D (nmol/L)	0.00894451	0.00167287	0.017700	<0.0001 *
Folate (mcg)	Log of Folate (ng/mL)	0.00013989	0.00004935	0.00610	0.0126 *
Folate (mcg)	Log of RBC Folate (ng/mL)	0.00008127	0.00003731	0.005100	0.0458 *
Iron (mg)	Log of Ferritin (ng/mL)	−0.00446666	0.00512466	0.002400	0.3972
Iron (mg)	Log of Hematocrit (%)	0.00117054	0.00025476	0.012300	0.0004 *
Iron (mg)	Log of Hemoglobin (g/dL)	0.00121445	0.00024645	0.011800	0.0002 *
Iron (mg)	Log of TIBC (µg/dL)	0.00265998	0.00235819	0.002400	0.2770
Copper (mg)	Log of Copper (mcg/dL)	−0.02984734	0.01001577	0.009600	0.0093 *
Phosphorus (mg)	Log of Alk Phos (IU/L)	0.00002507	0.00002507	0.001100	0.3110
Calcium (mg)	Log of Alk Phos (IU/L)	0.00006356	0.00003574	0.005600	0.0956
Protein (g)	Log of Albumin (g/dL)	0.0001764	0.0001764	0.013200	0.0261 *
Protein (g)	Log of BUN (mg/dL)	0.00121534	0.00023491	0.033000	0.0001 *
Protein (g)	Log of Total Protein (g/dL)	0.00002379	0.00005716	0.000300	0.6832

* Denotes significant when *p* < 0.05: RBC: red blood cell; TIBC: total iron-binding capacity; BUN: blood urea nitrogen.

## Data Availability

The data presented in this study are available in NHANES at https://wwwn.cdc.gov/nchs/nhanes/continuousnhanes/default.aspx?BeginYear=2015.
